# Protective effect of small molecule analogues of the *Acanthocheilonema viteae* secreted product ES-62 on oxazolone-induced ear inflammation

**DOI:** 10.1016/j.exppara.2015.03.025

**Published:** 2015-11

**Authors:** Lamyaa Al-Riyami, David T. Rodgers, Justyna Rzepecka, Miguel A. Pineda, Colin J. Suckling, Margaret M. Harnett, William Harnett

**Affiliations:** a Strathclyde Institute of Pharmacy and Biomedical Sciences, University of Strathclyde, Glasgow G4 0RE, UK; b Institute of Infection, Immunity and Inflammation, University of Glasgow, Glasgow G12 8TA, UK; c Department of Pure & Applied Chemistry, University of Strathclyde, Glasgow G1 1XI, UK

**Keywords:** ES-62, Immunomodulation, Oxazolone, Parasitic worm, Skin inflammation

## Abstract

ES-62 is the major secreted protein of the rodent filarial nematode *Acanthocheilonema viteae*. The molecule contains covalently attached phosphorylcholine (PC) residues, which confer anti-inflammatory properties on ES-62, underpinning the idea that drugs based on this active moiety may have therapeutic potential in human diseases associated with aberrant inflammation. Here we demonstrate that two synthetic small molecule analogues (SMAs) of ES-62 termed SMA 11a and SMA 12b are protective in the oxazolone-induced acute allergic contact dermatitis mouse model of skin inflammation, as measured by a significant reduction in ear inflammation following their administration before oxazolone sensitisation and before oxazolone challenge. Furthermore, it was found that when tested, 12b was effective at reducing ear swelling even when first administered before challenge. Histological analysis of the ears showed elevated cellular infiltration and collagen deposition in oxazolone-treated mice both of which were reduced by treatment with the two SMAs. Likewise, the oxazolone-induced increase in IFNγ mRNA in the ears was reduced but no effect on other cytokines investigated was observed. Finally, no influence on the mast cell populations in the ear was observed.

## Highlights

• Small molecule analogues (SMAs) of the immunomodulator, ES-62, have been produced.
• Two SMAs protect against oxazolone-induced skin inflammation in mouse ears.
• Protection is associated with reduced cellular infiltration and collagen deposition.
• Protection is associated with decreased IFNγ mRNA in the ears.

## Graphical Abstract

**Figure:**
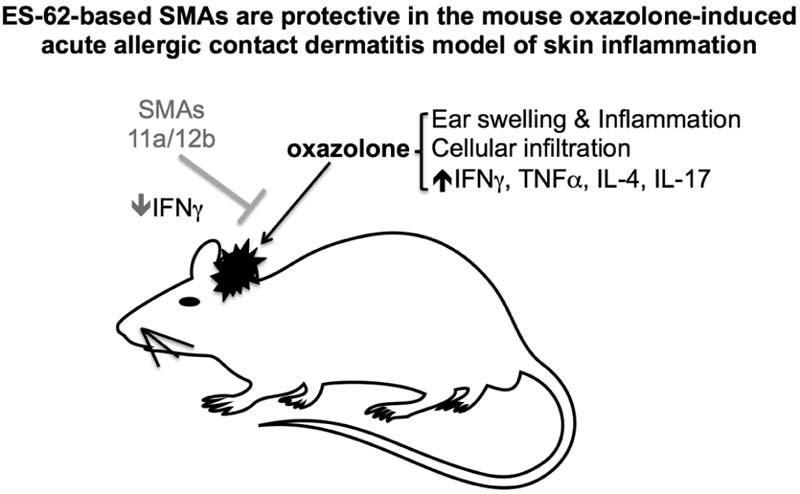

## 1 Introduction

A major cause of occupational skin disease is allergic contact dermatitis, which accounts for around 20% of all work-related health complaints. The mouse oxazolone contact hypersensitivity model is an experimental model system, which is employed to study human allergic contact dermatitis and indeed, has provided the framework for understanding of the human disease (Jin et al, 2009, Kaplan et al, 2012, Lundberg et al, 2012).

ES-62 is a glycoprotein secreted by the parasitic rodent filarial nematode *Acanthocheilonema viteae* (reviewed in Pineda et al., 2014). Its anti-inflammatory properties led to it being tested in both the collagen-induced arthritis (CIA) model of rheumatoid arthritis (Harnett et al, 2008, McInnes et al, 2003, Pineda et al, 2012) and the ovalbumin-induced airway hypersensitivity (OAH) model of asthma (Melendez et al, 2007, Rzepecka et al, 2013) where in both cases it was found to protect against disease development. That the molecule's ability to modulate disease is dependent on its unusual post-translational attachment of phosphorylcholine (PC) (reviewed in Harnett et al, 2010, Pineda et al, 2014) is supported by PC conjugated to ovalbumin (Harnett et al., 2008) or albumin (Al-Riyami et al., 2013) being able to mimic ES-62 in protecting against CIA. Subsequently, a library of drug-like small molecule analogues (SMAs) based around PC was developed, and two of them termed 11a and 12b (for structures, see Al-Riyami et al., 2013) have been shown to be protective in the CIA (Al-Riyami et al., 2013; unpublished) and OAH (Rzepecka et al., 2014) models. In this study, we employed the oxazolone-induced acute allergic contact dermatitis mouse model to test the ability of SMAs 11a and 12b to modulate skin inflammation. We now show that both 11a and 12b are effective at reducing ear swelling and this is associated with a clear reduction in cellular infiltration and collagen deposition as shown by histological analysis.

## 2 Materials and methods

### 2.1 Chemical synthesis and preparation of SMAs

Endotoxin-free SMAs 11a and 12b were prepared to ≥95% purity by HPLC and ^1^H NIMR as described previously (Al-Riyami et al., 2013). The SMAs were reconstituted at 100 mg/ml in sterile dimethyl sulfoxide (DMSO; Sigma-Aldrich) and then diluted in sterile PBS. Compounds were filter-sterilised using a Millex-GP (0.22 µm; Millipore) filter unit.

### 2.2 Oxazolone model

Animals were maintained and experimentation was undertaken in the Biological Services Unit with the approval of and in accordance with the Home Office UK and the Ethics Review Board of the University of Strathclyde.

On day 0, the ears of 10-week-old BALB/c mice (5/6 per group) were sensitised with 1% oxazolone (10 µl on each side; 4-ethyoxymethylene-2-phenyloxazolone; Sigma-Aldrich) in acetone:sesame seed oil (4:1). On days 5 and 6, 1% oxazolone (20 µl) was applied to the inner and outer surfaces of the right ear and the same amount of solvent alone to the left ear. Ear thickness was measured using a calliper before and 1 h and 24 h after challenge. To ensure that the swelling was due to oxazolone-specific inflammation, a non-sensitised but challenged control group was included. SMA 11a (1 µg), 12b (1 µg) or PBS was injected on days −1, 4 and 5 or day −1 only or days 4 and 5 only, subcutaneously. The dose of 1 µg of SMA was chosen as it was previously shown to be effective in preventing pathology in CIA (Al-Riyami et al., 2013). Animals were sacrificed and the ears harvested on day 6, 1 h after challenge. For histological analysis, the ears were fixed in 10% formalin for at least 24 h. For mRNA analysis, ears were kept in RNA-Later (Qiagen) and stored at −20 °C.

### 2.3 *Ex vivo* analysis

#### 2.3.1 Histology

Formalin-fixed ears were embedded in OCT medium (Tissue Tek) and snap frozen. Tissue sections (7 µm) were then cut using a cryotome (Fisher Scientific) and stained with haematoxylin and eosin (H&E) to show cellular infiltration and tissue structure, Gomori trichrome to show collagen deposition, and toluidine blue to stain mast cells in the tissue.

#### 2.3.2 qRT-PCR

Total RNA was extracted using an RNeasy Fibrous kit (Qiagen) and ≤1 µg of RNA was used to synthesise cDNA (Applied Biosystems). TaqMan^®^ RT-PCR was performed using the following TaqMan^®^ Gene Expression Assays: IL-17A (Mm00439619_m1), IFNγ (Mm01168134_m1), IL-4 (Mm00445259_m1), IL-22 (Mm01226722_g1), TNFα (Mm00443259_g1) and IL-6 (Mm00446190m1), all from Applied Biosystems. Polymerase chain reactions were performed in duplicate in a StepOne sequence detector (Applied Biosystems). Data analysis was performed using the Applied Biosystems sequence detection software and samples were normalised to the reference reporter mouse glyceraldehyde 3-phosphate dehydrogenase (GAPDH; Mm99999915_g1) endogenous control.

### 2.4 Statistics

Data were analysed by Student's t test where *p < 0.05, **p < 0.01 and ***p < 0.001.

## 3 Results

**Fig. 1:**
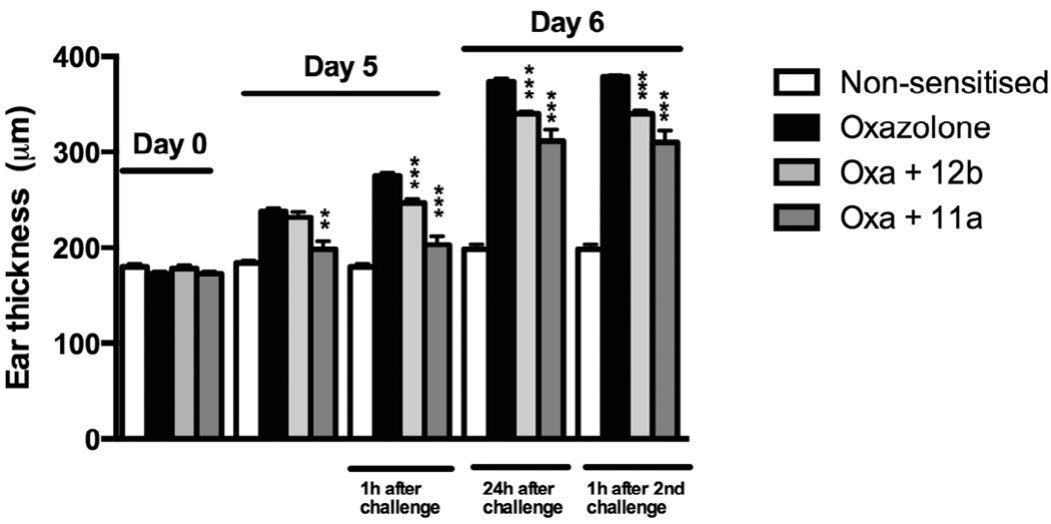
The effect of treatment with 1 µg of SMAs 12b and 11a on days −1, 4 and 5 on ear thickness was assessed following sensitisation and challenge with oxazolone. A non-sensitised control group was also included. The data are obtained from a single experiment, which is representative of two (11a) and three (12b) independent experiments. **p < 0.01, ***p < 0.001. ** and *** represent a significant difference between 12b − (Oxa + 12b) or 11a − (Oxa + 11a) treated and PBS-treated mice sensitised and challenged with oxazolone (Oxazolone). Results are expressed as ear thickness in micrometres.

Oxazolone-induced ear inflammation – a well-characterised model of human atopic dermatitis involving sensitisation to and challenge with oxazolone (Jin et al, 2009, Kaplan et al, 2012, Lundberg et al, 2012) was employed for testing of the SMAs as described in Section 2. SMAs 11a and 12b both reduced oxazolone-induced ear swelling observed after challenge on days 5 (1 h after challenge) and 6 (24 h after day 5 challenge and  h after day 6 challenge), compared to the PBS-treated oxazolone group, when administered on days −1, 4 and 5 (Fig. 1). Moreover, the protective effects of 11a were observed on day 5 prior to challenge with the ear swelling being significantly reduced (31.3 ± 1.38% of the increase in ear thickness observed in the oxazolone group relative to the non-sensitised control). In detail, reduced increases in ear thickness were observed, both 1 h (11a; 24.53 ± 1.06%; 12b: 70.21 ± 1.19% of the increase in oxazolone group relative to the non-sensitised control) and 24 h (11a: 68.1 ± 2.54%; 12b: 82.8 ± 0.63% of the increase in oxazolone group relative to the non-sensitised control) following the first challenge on day 5, and ears from the animals treated with 11a (65.6 ± 2.56% of the increase in oxazolone group relative to the non-sensitised control) and 12b (80.6 ± 0.86% of increase in oxazolone group relative to the non-sensitised control), after the second challenge on day 6 also remained significantly reduced in size compared to the oxazolone group (Fig. 1). The data shown are obtained from a single experiment, which is representative of a number of independent studies employing 11a (n = 2) and 12b (n = 3).

Additionally, treatment with SMA 12b before sensitisation on day −1 only was also tested in two separate experiments. 12b was able to significantly reduce ear swelling 1 h after the first challenge on day 5 (pooled data; oxazolone group: 275.8 ± 2.6 µm (SEM), n = 12; oxazolone + SMA 12b group: 246.4 ± 6.6 µm, n = 11; p < 0.001) but was unable to maintain that reduction 24 h after challenge and following the second challenge, no reduction in ear swelling was witnessed either (results not shown). Treatment with 12b only before challenge on days 4 and 5 only was also undertaken, but 12b was not able to consistently reduce ear swelling under this protocol (results not shown).

**Fig. 2:**
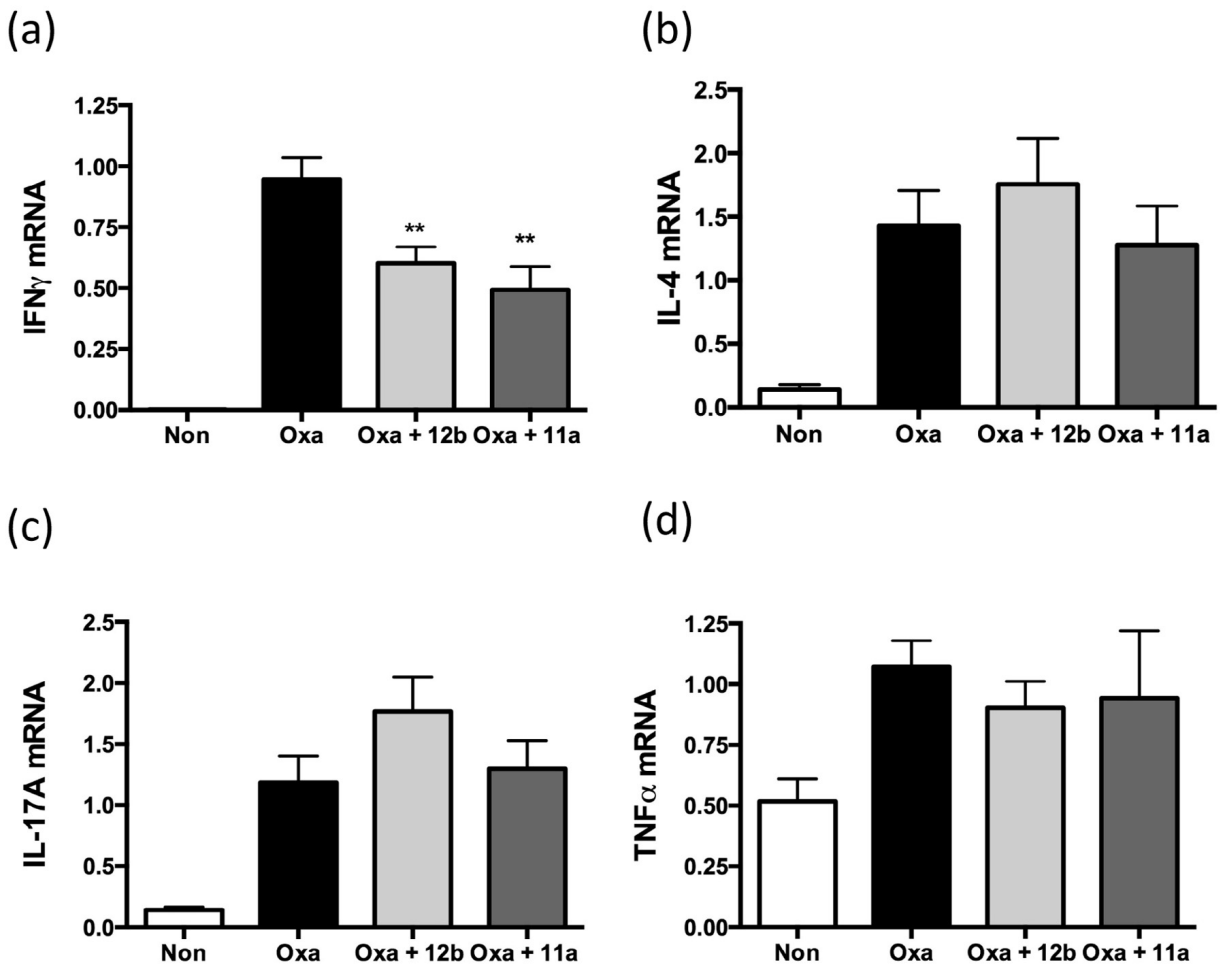
The effect of treatment with 1 µg of SMAs 11a and 12b on days −1, 4 and 5 on mRNA levels of IFNγ (a), IL-4 (b), IL-17A (c) and TNFα (d) from day 6 ears, 1 h after challenge, as assessed by qRT-PCR where the levels of the gene of interest were normalised to the level of GAPDH. Data are presented as the means ± SEM of the mean of replicate values pooled from 3 individual experiments. **p < 0.01. **Significant difference between 12b- (Oxa + 12b) or 11a- (Oxa + 11a) treated and PBS-treated mice sensitised and challenged with oxazolone (Oxa). The non-sensitised group is represented by “non”.

Elevated levels of IFNγ, IL-4, IL-17A and TNFα mRNA were witnessed in the oxazolone, compared to the non-sensitised group (Fig. 2a–d). By contrast, there was no difference in the mRNA expression levels of IL-6 and IL-22 observed in the oxazolone-treated compared to the non-sensitised group (results not shown). Treatment with SMAs 11a and 12b (both at 1 µg) on days −1, 4 and 5 caused a significant down-regulation of IFNγ mRNA (Fig. 2a). By contrast, treatment with 11a and 12b did not have any significant effect on the expression levels of IL-4, IL-17A or TNF-α (Fig. 2b–d). Likewise, the SMAs were also found to have no effect on the steady-state levels of IL-6 or IL-22 (only 12b tested) detected (results not shown).

**Fig. 3:**
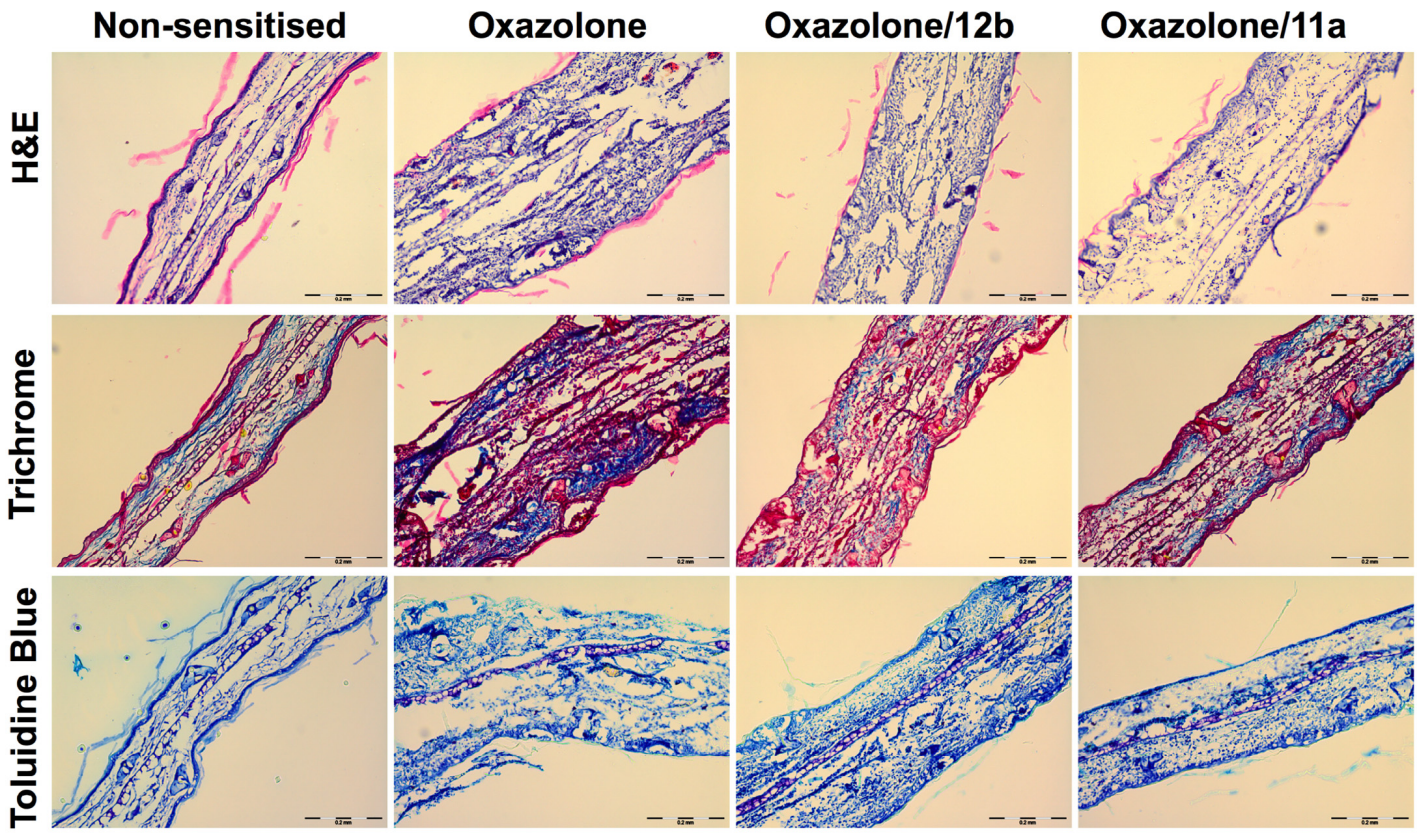
Ear sections from non-sensitised, oxazolone and oxazolone/SMA-treated (days −1, 4 and 5) mice were stained with haematoxylin and eosin (H&E) to show cellular infiltration and tissue structure, Gomori trichrome to show collagen deposition, and toluidine blue to stain mast cells in the tissue.

Histological analysis of the ears showed elevated cellular infiltration and collagen deposition in oxazolone-treated mice that was reduced by treatment with the two SMAs (Fig. 3). No clear difference in the mast cell populations in the ear was observed amongst any of the groups as indicated by staining by toluidine blue (Fig. 3).

## 4 Discussion

It is generally accepted that ES-62 is amongst the best characterised of helminth-derived immunomodulators (Al-Riyami, Harnett, 2012, Pineda et al, 2014). It has been shown to be active in mouse models of both allergic, including oxazolone-induced contact hypersensitivity (Melendez et al., 2007), and autoimmune diseases, and has been described as the helminth-derived molecule with most potential for testing in humans for treatment of such disorders (Erb, 2009). However, ES-62 is a large protein, and therefore potentially immunogenic, thus making it an unreliable drug candidate. However, the data we have acquired over a number of years indicated that the active component of ES-62 is its PC moiety and we therefore subsequently designed a library of synthetic, small, drug-like compounds based around PC (Al-Riyami et al., 2013). Both SMA 11a and SMA 12b are too small to be immunogenic and hence are ideal candidates for more drug-like versions of ES-62 for therapeutic purposes. In this study, we used the mouse model of immediate-type hypersensitivity to oxazolone to test the activity of SMAs 11a and 12b against skin inflammation.

We clearly demonstrated the ability of both 11a and 12b to protect against ear swelling when administered on days −1, 4 and 5. We then further explored the protective effects and histological examination of ear sections from treated animals revealed reduced levels of cellular infiltration and collagen deposition. The significance of mast cells during contact hypersensitivity has been reported to be quite controversial (Kaplan et al., 2012), with conflicting reports regarding their role. Thus, some authors have reported an essential role for mast cells in contact hypersensitivity whilst others reported that mast cells were redundant for this (Askenase et al, 1983, Galli, Hammel, 1984). In this present study, following histological examination, there was no clear difference observed in the mast cell numbers in the ears when comparing oxazolone-treated, oxazolone/SMA-treated and control samples. Their numbers were generally low and this contributed to it being difficult to determine whether the SMAs are having an effect on mast cell degranulation. A failure to find such an effect would differ from our previous work employing ES-62 in the model (Melendez et al., 2007). However, despite certain of our data obtained to date suggesting that ES-62, PC-conjugates and SMAs may employ the same mechanism of action (Al-Riyami et al, 2013, Rzepecka et al, 2014) some previous data employing PC conjugates indicate that this might not always be the case (Harnett et al., 2008). In any case, although it appears in the present study that the SMAs are working in a mast cell-independent manner, this does not exclude them from suppressing mast cell responses in other situations in which these cells may play a role.

Pro-inflammatory cytokines such as TNFα, IL-4, IL-6, IFNγ and increasingly, IL-17 (Cosmi et al, 2014, Incorvaia et al, 2008, McFadden et al, 2013, Taniguchi et al, 2013, Watanabe et al, 2002) are likely to play an important role in allergic contact dermatitis and other inflammatory skin conditions. For example, Webb et al. demonstrated up-regulated levels of TNFα, with a significant increase at 4 h in the oxazolone-induced acute allergic contact dermatitis model. They also showed that IFNγ increased significantly at 24 h (Webb et al., 1998). In our study, when examining mRNA levels, a strong induction of IFNγ, IL-4, IL-17A and TNFα expression was seen after oxazolone challenge, consistent with the previous findings reported for this model (Ahlfors, Lyberg, 2010, Fujii, Sengoku, 2013, Heo et al, 2015, Webb et al, 1998). However, only a significant reduction in IFNγ levels was witnessed in mouse ears when treatment with SMA 11a or 12b was undertaken. Of note, a similar reduction in release of the signature Th1 cytokine IFNγ was exhibited when 11a was tested in CIA (Al-Riyami et al., 2013) and when peripheral blood mononuclear cells and synovial fluid membrane cultures from human patients with RA were pre-exposed to ES-62 (Harnett et al., 2008).

In summary, we find that SMAs of ES-62 can be effective in ameliorating skin inflammation in a mouse model. Taken together our results suggest that this study represents a good starting point for the design of a novel class of drugs, which could potentially be used to treat allergic contact dermatitis.

## Author's contribution

LA, DR, JR and MAP planned and performed experiments and WH and MMH planned and supervised the study. CJS designed and developed the ES-62 SMAs. All authors contributed to the analysis of the data and preparation of the manuscript.

## Conflict of interest

The authors have no conflict of interest.
